# Recent progress of porcine milk components and mammary gland function

**DOI:** 10.1186/s40104-018-0291-8

**Published:** 2018-10-22

**Authors:** Shihai Zhang, Fang Chen, Yinzhi Zhang, Yantao Lv, Jinghui Heng, Tian Min, Lilang Li, Wutai Guan

**Affiliations:** 1 Guangdong Province Key Laboratory of Animal Nutrition Control, College of Animal Science, South China Agricultural University, Guangzhou, 510642 China; 2 College of Animal Science and National Engineering Research Center for Breeding Swine Industry, South China Agricultural University, Guangzhou, 510642 China; 3 Agro-Biological Gene Research Center, Guangdong Academy of Agricultural Sciences, Guangzhou, 510640 China

**Keywords:** Bioactive components, Fat, Lactose, Mammary gland, Porcine milk, Protein

## Abstract

As the only nutritional source for newborn piglets, porcine colostrum and milk contain critical nutritional and immunological components including carbohydrates, lipids, and proteins (immunoglobulins). However, porcine milk composition is more complex than these three components. Recently, scientists identified additional and novel components of sow colostrum and milk, including exosomes, oligosaccharides, and bacteria, which possibly act as biological signals and modulate the intestinal environment and immune status in piglets and later in life. Evaluation of these nutritional and non-nutritional components in porcine milk will help better understand the nutritional and biological function of porcine colostrum and milk. Furthermore, some important functions of the porcine mammary gland have been reported in recent published literature. These preliminary studies hypothesized how glucose, amino acids, and fatty acids are transported from maternal blood to the porcine mammary gland for milk synthesis. Therefore, we summarized recent reports on sow milk composition and porcine mammary gland function in this review, with particular emphasis on macronutrient transfer and synthesis mechanisms, which might offer a possible approach for regulation of milk synthesis in the future.

**Electronic supplementary material:**

The online version of this article (10.1186/s40104-018-0291-8) contains supplementary material, which is available to authorized users.

## Background

Piglets are born with low body fat reserves and immature immune systems. Timely colostrum intake is crucial for piglets to gain sufficient nutrients and passive immunoglobulins from the sow after piglets are exposed to environmental cold and pathogens [1]. Mei et al. [2] reported that 200 g of colostrum per piglet during the first 24 h after birth could reduce the risk of mortality before weaning. Furthermore, porcine milk has been shown to play an essential role in enhancing piglet performance [3] and stimulate visceral organ and skeletal muscle protein synthesis in neonatal piglets [4].

Traditionally, porcine milk has been considered to be mainly composed of carbohydrates, lipids, and proteins (immunoglobulins) with small proportions of minerals, vitamins, leukocytes and somatic cells. However, recently studies reported additional components in porcine milk. Scientists have identified novel components of sow colostrum and milk, including exosomes, oligosaccharides, and bacteria [5–7]. These recently reported components play a possible vital role in stimulating development of the neonatal immune system and establishing intestinal bacterial communities. Since total piglets born has increased from 12 to 14 to 14–16 per farrowing over the last few decades, it is important that sufficient essential nutrients for the piglets be produced by the prolific sow. Therefore, a systematic review regarding empirical and novel components of porcine milk will help to better understand the nutritional and biological functions in production of milk by sows.

Different from porcine milk, bovine milk is a biological fluid used for human food. The function of the bovine mammary gland has been well studied for many years. However, unlike bovine lactation, some of the important functions of the porcine mammary gland have been only recently reported in published literatures, such as: 1) mechanisms for glucose transfer into the mammary gland and subsequent synthesis of lactose; 2) the potential amino acid transporters in the porcine mammary gland for amino acid accumulation; and 3) fatty acid synthesis in the porcine mammary gland. Therefore, we summarized the recent research progress in porcine mammary gland function in this review, especially macronutrient (lactose, protein and fatty acids) transfer and synthesis mechanisms. The effects of nutrition, hormones, and environment on porcine milk synthesis have been thoroughly reviewed by Farmer and Quesnel [8], and will not be discussed in this review.

## Composition of protein, fat, and lactose in sow milk

**Fig. 1:**
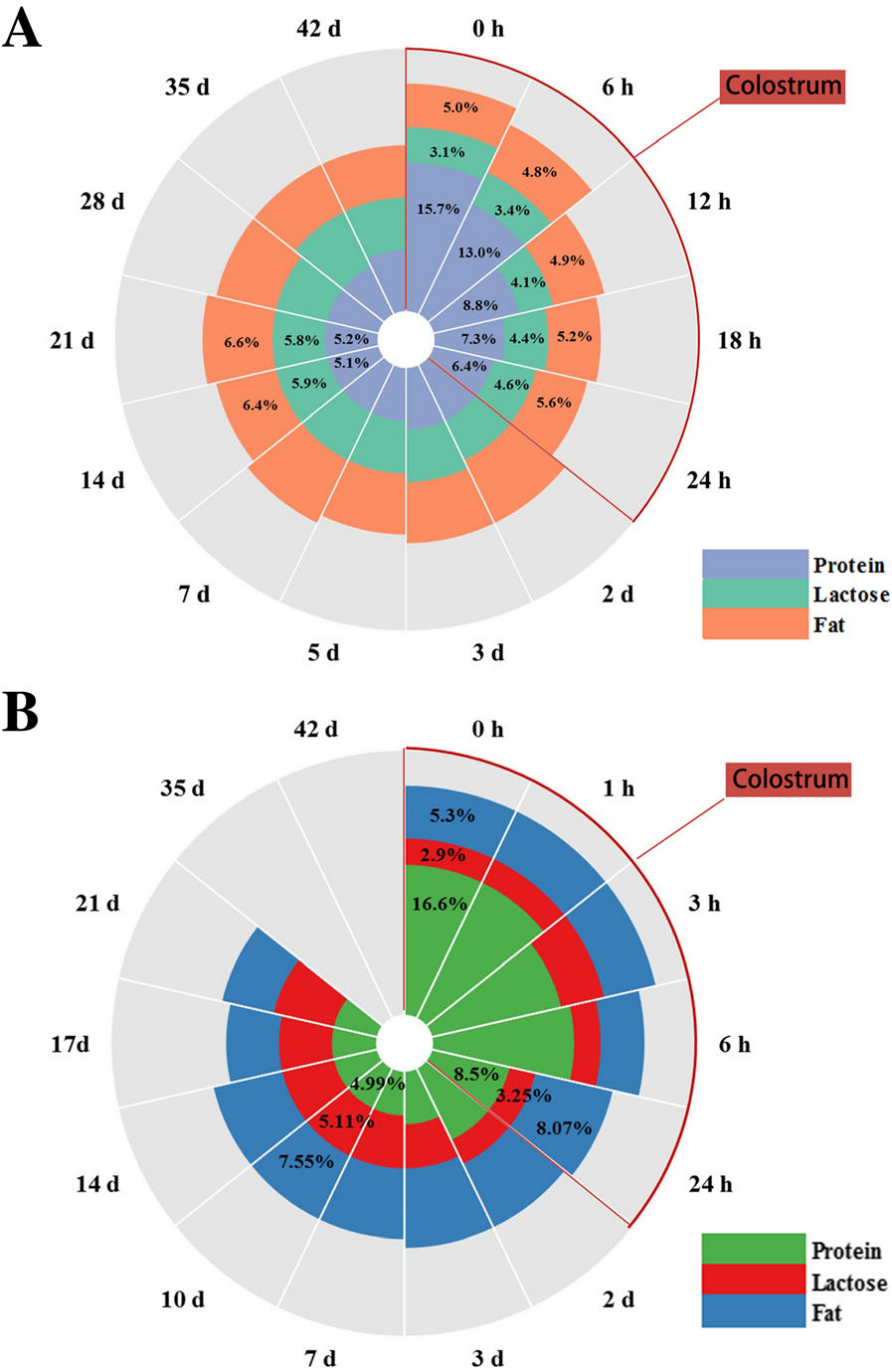
Composition of sow milk throughout lactation. Note: **a**. Porcine milk composition in 1980s. These data were summarized by Klobasa et al. [103]. **b**. Porcine milk composition in 2010s. References used to calculate averages of milk composition in last 10 years: Kim et al. [104], Tian et al. [105], Bai et al. [106], Wang et al. [107], Shen et al. [108], Decaluwe et al. [109], Wang et al. [110], Samanc et al. [111], Loisel et al. [112], Krogh et al. [113], Flummer and Theil [114], Zhao et al. [115], Velayudhan and Nyachoti [116], Rosero et al. [117], Farmer et al. [118], Park et al. [119], Wang et al. [120]

Similar to other mammals, protein, fat, and lactose are the three predominant components in sow milk (except water). Previously, Jensen [9] thoroughly summarized milk components of more than 30 mammals (Additional file 1: Table S1). The medians of protein, fat, and lactose concentrations among the different mammals are 5.85%, 5.4% and 4.6%, respectively. Sows appear to have comparatively higher milk fat concentration than the median mammal level, but not milk protein or lactose. With the recent enhancement of reproductive performance in sows, we hypothesized higher level of macronutrient components in porcine milk. However, milk macronutrient components do not significantly increase with enhanced reproductive performance in sows as depicted in Fig. 1; current concentrations of protein, fat and lactose in colostrum are similar to those of 30 years ago (16% protein, 3% lactose and 5% fat) (as shown in Additional file 1: Table S2). Only minor changes in sow milk have been observed over the past 30 years (as shown in Additional file 1: Table S3). Recent research has reported relatively higher levels of fat (7.5% vs. 6.5%), lower levels of lactose (5% vs. 6%) and similar protein expression (5%). It was demonstrated that litter size does not regulate total sow milk yield using ‘weigh–suckle–weigh’ estimates of milk yield [10]. This indicates that nutrient intake of piglets from large litters can be restricted, as they receive less milk with similar quality. Therefore, provision of milk replacer for neonates in prolific sows might be an effective way to sustain their survival rate and weaning weight.

## Progress in porcine milk components

### Porcine milk oligosaccharides

Porcine milk oligosaccharides (PMOs) are composed of 3–10 monosaccharides bonded through glycosidic bonds. PMOs are not easily digested and they have anti-infective and prebiotic functions in piglets [11–13]. Milk oligosaccharides are widely found in humans (more than 200 determined and 115 identified) and bovines (about 40 determined and 25 identified) [14–18]. PMOs in porcine milk have received little attention until recently. Tao et al. [5] identified nearly 30 different PMOs in porcine milk. They further demonstrated that the prevalent PMO in porcine milk is sialylated oligosaccharides, most containing 3–8 monosaccharides (e.g., glucose, galactose, N-acetylglucosamine, N-acetylneuraminic acid, and N-glycolylneuraminic acid). Recently, 13 and 25 novel PMOs in sow milk were identified by Difilippo et al. [19] and Wei et al. [20], respectively. Currently, more than 90 oligosaccharides in sow milk have been reported [20]. PMOs can be divided into neutral-, sialyl- and fucosyl-PMOs. Sialylated and neutral PMOs appear to be the dominant PMOs in sow milk, especially in colostrum [5, 20]. The variation in specific PMOs among different studies might be associated with different test methods. As presented in Table 1, neutral PMOs accounted for about 70–80% of total PMOs in Yorkshrie, while sialylated PMOs were the dominant PMOs (60–80% of total PMOs) in Landrace. Although the data are still quite limited and obtained in different survey for the different breeds, these reports indicate PMOs could be significantly variable among different breeds.

**Table 1:** Porcine milk oligosaccharides of Yorkshire and Landrace in colostrum and milk (%)

Breeds	Source	Neutral OS	Acidic-sialylated OS	Neutral-fucosylated OS	References
Yorkshire	Colostrum	69	16	2	[121]
	Transitional milk	76	19	3	
	Mature milk	81	29	4	
Landrace	Colostrum	22	78	1	[20]
	Transitional milk	18	74	7	
	Mature milk	33	58	9	

PMO content was reported highest in colostrum, gradually decreasing during early lactation, but increased at 24 d [5]. Furthermore, proportions of specific PMOs change with the progress of lactation. PMOs in porcine milk are parity dependent and more abundant in the gilt (females pig who has not farrowed or given birth to a litter previously) than the sow (females pig who has farrowed or given birth to a litter previously). High concentrations of PMOs might partly compensate for deficiencies in milk volume in colostrum [20]. Furthermore, high PMO concentrations in the mammary gland might protect the gilt mammary gland from bacterial infection. Although PMOs are resistant to digestion in the small intestine, they are fermentable substrates in the large intestine, which is evident because few PMOs are found in the feces of neonatal piglets [19]. These oligosaccharides can be fermented into short chain fatty acids (SCFAs) including butyric acid which has been widely demonstrated to participate in regulation of mucosal immunity, gut integrity, and colonic health [21–23]. PMOs can also prevent pathogens from adhering to the mucosa since they contain analogues to various receptors for microbes [24, 25]. These oligosaccharides also have been reported to selectively stimulate growth of beneficial bacteria [11, 12], which might be related to their fucosylated and sialylated structures [13]. Thus, oligosaccharides appear to have beneficial biological functions for piglets. Future studies need to clarify whether artificial oligosaccharides can be used as feed additives in the weaning pig diet. Currently, limited data are available on the impact of the sow genotype on PMO composition in colostrum and milk. A better understanding of these variations is vitally important for efficient pig production. Furthermore, mammary glands are susceptible to infection during lactation and the relationship of PMOs to immunity from mammary infection is largely unknown, suggesting a need for future research.

### Exosomes

Extracellular vesicles are a heterogeneous group of cell-derived membranous structures comprised of exosomes and microvesicles, which are present in biological fluids and involved in multiple physiological processes [26]. Exosomes, nanosized endosome-derived membrane vesicles (40–100 nm in diameter), were found in porcine milk in recent studies [6, 27]. These vesicles contain mRNA, microRNA (miRNA), DNA, proteins, and lipids and can possibly transfer these bioactive components to neonatal pigs [28–30]. It has been reported that 16,304 mRNAs, 237 miRNAs and 639 proteins have been identified in porcine milk exosomes by RNA-sequencing and proteomic analysis and predicted to be involved in immunity, proliferation and cellular signaling [30]. miRNAs have been the predominant focus of most recent research. Milk miRNAs in exosomes are stable to intestinal digestion and permeable to the intestinal barrier [31]. Although the functions of exosomes are still controversial, some studies hypothesized that exosomal miRNAs could be transferred from maternal milk to neonates via the digestive tract, participating in regulating the neonatal immune system [27, 32] and simulating gastric/pancreatic digestion [33]. Studies focusing on the function of exosomes in porcine milk are very limited. Chen et al. [34] found porcine milk exosomes can regulate intestinal cell proliferation and digestive tract development. However, whether exosomes have an important role in neonatal growth and regulate neonatal immunity needs to be verified in in vivo models. It is apparent that our knowledge regarding porcine milk exosomes is quite limited. Future studies need to clarify the following: stability of the components of porcine milk exosomes through lactation (colostrum, transitional milk and mature milk); identification of core functional components of porcine milk exosomes in addition to miRNAs; and regulation of porcine milk exosomes through nutrition, conventional breeding, and genetics.

### Bacteria in milk

Milk has been identified as an excellent source of probiotic lactic acid bacteria for neonates in humans and cows [35–37]. These lactic acid bacteria might originate from the maternal intestine, translocate to breast milk through the bloodstream, and subsequently colonize the gastrointestinal tract of neonates [38]. Recently, Martin et al. [7] reported some strains displayed high probiotic potential in sow milk, such as *Lb. reuteri* CR20 (a reuterin-producing strain), *Lb. salivarius* CELA2 (a bacteriocin-producing strain) and *Lb. paraplantarum* CLB7. However, it is well-known that milk is also a good culture medium for bacteria from environmental sources. For example, the teat surface can contain a high diversity of bacteria [39, 40]. Therefore, existence of the bacterial entero-mammary pathway needs further confirmation. Further research needs to clarify whether the source of bacteria is from the sow, environmental sources, or both. However, there is potential for beneficial bacteria in milk to be developed into a feed additive for weaning piglets.

### Leukocytes in milk

Traditionally, it has been thought that immunoglobulins in colostrum are the only source of adaptive immunity from sow to piglets. However, leucocytes in sow milk can also be a source of immunity. The average proportion of leucocytes in colostrum is 1 × 10^7^ cells/mL, which decreases to 1 × 10^6^ cells/mL in sow milk [41], similar to human milk [42]. In sow milk, leucocytes are primarily composed of neutrophils, macrophages and lymphocytes, common components in milk. Neutrophils have been regarded as the final effector cells of an acute inflammatory response and mainly participate in the clearance of extracellular pathogens. Research has demonstrated that neutrophils are involved in the activation, regulation, and effector functions of innate and adaptive immune cells [43]. Recently, lymphocyte subsets in sow milk have been identified by Pomorska-Mol et al. [44], consisting mainly of T-lymphocytes, B-lymphocytes, T-helper lymphocytes, and T-cytotoxic lymphocytes. Activated T cells from sows could compensate for the immature function of neonatal T cells and promote their maturation [45]. Macrophages are widely known for phagocytic activity and also participate in regulation of the function of neonatal T and B cells and in secretion of immunoregulatory factors [46]. However, even though leukocytes exist in milk, the phagocytic capacity of colostrum and milk leukocytes was reported to be weak compared with blood leukocytes [47]. Thus, leukocytes in porcine milk are more likely important immunity regulators rather than phagocytic cells.

### Hormones in milk

Hormones existing in sow blood can be transported and secreted into milk through the mammary gland. These bioactive compounds in milk can play important roles in mammary cell regulation and neonatal function (gastrointestinal tract or systemic) [48]. IGF-I and IGF-II are present in both sow colostrum and milk. Concentrations of IGF-I have been reported as 136 ng/mL and 10–14 ng/mL in colostrum and milk, respectively [49]. Published estimates of IGF-II levels are higher than IGF-I with values of 291 ng/mL and 11–29 ng/mL in colostrum and milk, respectively [49]. Correspondingly, IGF binding proteins (IGFBP) are also present in porcine milk [49]. Another hormone found in porcine milk is insulin. Similar to IGF-I and IGF-II, the concentration of insulin is high at the beginning of lactation (about 400 μU/mL), then decreases to approximately 30 μU/mL [50]. Also, recent studies have found that relaxin is delivered from mother to offspring via the consumption of colostrum (9–19 ng/mL) and milk (2 ng/mL, postnatal 14 d) [51, 52]. These observations support a role for relaxin as a lactocrine mediator in the development of the neonatal pig. Other growth-stimulating factors, such as epidermal growth factor (EGF), have also been detected in sow milk [53].

Hormone receptors for insulin, prolactin and IGF-I were reported to be expressed in the porcine mammary gland, which further demonstrates that these hormones have important biological functions in the mammary gland [54]. Despite the potential vital functions of hormones in milk, most of the research reports that focus on hormones in milk are from approximately 20 years ago. This may be because hormones are relatively low in milk compared with those in blood and not considered metabolically important. Secondarily, it is difficult to detect hormones in milk and especially colostrum because of their viscosity and a large number of hormones are binding to casein. However, it is important to not ignore the function of hormones in milk, because they can play a vital role even in small concentrations.

### Other metabolites

Recently, 25 metabolites were identified in colostrum, including monosaccharides, disaccharides (such as lactose), organic acids (lactate, citrate, acetate and formate), nitrogenous organic acids (such as creatine) and other compounds (including nucleotides) [55]. Similar to porcine milk glycemics, it was reported that colostrum metabolome is greatly affected by different breeds (colostrum composition of Duroc sows differ from Landrace and Large White sows) [55]. Interesting, this study found some biomarkers are positively-related (acetate and taurine) or negatively-related (dimethylamine and cis-aconitate) to litter weight gain [55].

## Recent progress in mammary gland function

### Glucose uptake and lactose synthesis

Glucose is an important precursor to some of the constituents of milk, despite low concentrations in sow milk. Approximately 59% of plasma glucose transported into the mammary gland contributes to lactose in sow milk, while the remaining glucose is converted into glycerol, casein, albumin, and oxidized into CO_2_ [56, 57]. Consequently, transportation of glucose into the mammary gland is a critical step in milk synthesis. Glucose transportation into the mammary gland is a complicated process requiring coordination of a variety of membrane proteins, but the specific proteins involved differ among species. In the bovine mammary gland, dominant glucose transporters are glucose transporter-1 (GLUT1) and GLUT8, and sodium-dependent glucose cotransporters 1 (SGLT1), SGLT2 and GLUT12 are also expressed. [58–60]. In rats, GLUT1, SGLT1, GLUT12 appear to play a vital role in glucose transport to the mammary gland [61, 62]. Compared with literature for bovine and rats, reports focusing on the glucose transport system of lactating sows are limited. Chen et al. [63] reported mRNA abundance for glucose transporters *GLUT1*, *GLUT8*, *SGLT1*, *SGLT3*, and *SGLT5* in the mammary gland. The mRNA abundance of *GLUT1* was at least 10 times more abundant compared to other glucose transporters with high protein expression [63]. Furthermore, a significant increase was reported in the mRNA level of *GLUT1* and *SGLT1* after sows progressed from gestation to peak lactation [63], which is consistent with other reports [64]. These studies indicate that GLUT1 could be the most important transporter in the porcine mammary gland. However, only mRNA levels of other glucose transposers are generally available in the literature. To clarify the specific function of each glucose transporter, future research is needed to evaluate the protein abundance of these glucose transporters and localize their position in the porcine mammary gland by immunofluorescence microscopy or confocal microscopy. Also, CRISP-Cas9 could be used to knock-out these transport genes and determine their effects on glucose transportation from plasma to cellular fluid or cellular fluid to Golgi bodies.

**Fig. 2:**
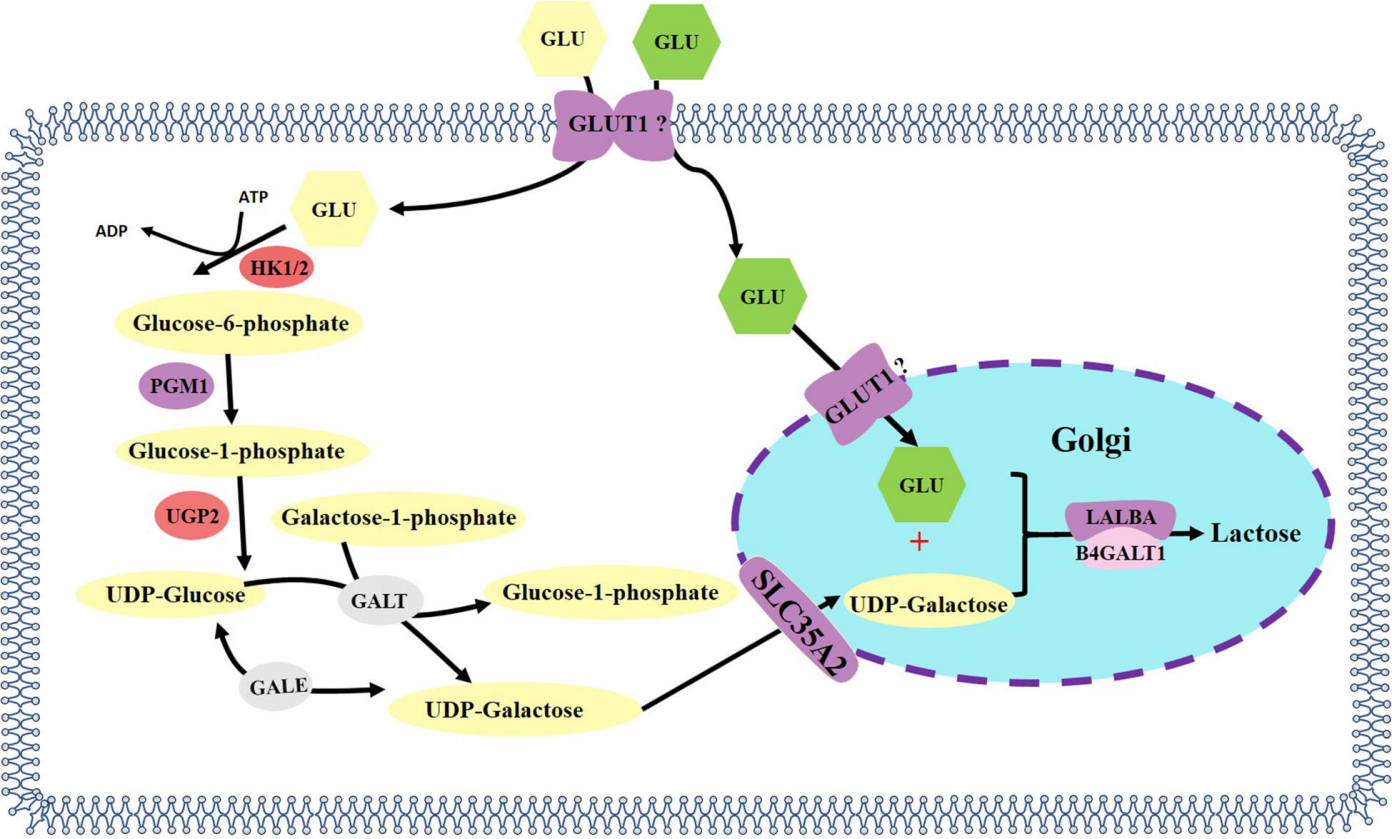
Lactose synthesis pathway in porcine mammary gland. Note: Glucose is taken up by the porcine mammary gland through membrane glucose transporters (possibly GLUT1). In the cytoplasm, after glucose is converted into glucose-6-phosphate, it will be further converted into UDP-galactose by a series of enzymes: PGM, UGP2, GALT, and GALE. Subsequently, glucose and UDP-galactose in the cytoplasm is transported into the Golgi bodies by GLUT1 (possibly) and SLC35A2, respectively. Finally, glucose and UDP-galactose are synthesized into lactose by LALBA and B4GALT1 in the Golgi bodies. After the initation of lactation, mRNA of enzymes is increased 2-fold or more are shown in light purple; those increasing 1.5-to1.9-fold are colored light red, and those expression increase or decrease less than 1.5-fold are shown in light gray. Abbreviation: GLUT1, glucose transporter-1; PGM, phosphoglycerate mutase; UGP2: UDP-glucose pyrophosphorylase 2; GALT, galactose-1-phosphate uridyltransferase; GALE, e UDP-glucose 4-epimerase gene; LALBA, alpha-lactalbumin gene; β-1,4-galactosyltransferase

Glucose is largely converted into lactose after it is transferred into the mammary gland. Lactose synthesis requires the coordination of many genes or proteins including phosphoglucomutase 1 (PGM1), UDP-glucose pyrophosphorylase 2 (UGP2), galactose-1-phosphate uridylyltransferase (GALT), and UDP-galactose-4-epimerase (*GALE*) (interconversion of glucose and galactose), *SLC35A2* (transportation of UDP-galactose into Golgi bodies), α-lactalbumin (*LALBA*) and β-1,4-galactosyltransferase-1 (*B4GALT1*) (under which glucose and UDP-galactose synthesize lactose) [65–67]. However, most reports in the literature focus on lactose synthesis in rat, bovine, goat and human models. In lactating sows, the critical genes regarding lactose synthesis have only recently been reported. Chen et al. [63] reported that the gene and protein expression of B4GALT1 significantly increased in sows’ mammary glands from late pregnancy to peak lactation. *B4GALT1* encodes type II membrane-bound glycoprotein, specifically β-1, 4-galactosyltransferase 1, lactose synthetase, which can transfer galactose to acceptor sugars [68]. Coincidently, Zhang et al. [69] also found the mRNA and protein expression profiles of B4GALT1 increased significantly during the entirety of lactation in sows Furthermore, the increased mRNA and protein expression of LALBA was also observed in their research. α-lactalbumin, one of milk whey proteins, can facilitate the glucose identification and banding of B4GALT1, and improve the B4GALT1 activity by at least 30-fold [70–73]. These research reports of lactose synthesis in sows indicated that *LALBA* and *B4GALT1* are the critical genes or steps in the lactose synthesis pathway of the sow. *LALBA* and *B4GALT1* are also highly conserved in humans [74], cows [75], goats [76] and other species*.* and have critical functions in lactose synthesis. Current knowledge about possible mechanisms of lactose synthesis in the porcine mammary gland is presented in Fig. 2**.**

### Amino acid uptake and protein synthesis

The mammary gland requires large amounts of amino acids for the synthesis of milk protein. Differences in amino acid concentrations in the veins and arteries of lactating mammary glands suggest that the mammary gland extracts essential amino acids primarily from the blood. Amino acid uptake by the porcine mammary gland can be divided into three levels: high level (dose > 30 g/d; leucine, arginine and lysine), middle level (15 g/d < dose < 30 g/d; valine, isoleucine, threonine, phenylalanine), and low level (dose < 15 g/d tyrosine and methionine and histidine) [77, 78]. Presently, the amino acid transporter system in the mammary gland is not well-understood, but information suggests that the mammary gland has a transport system similar to other organs (intestine, kidney, placenta). As presented in Table 2, the porcine mammary gland expresses cationic amino acid transporters (*CAT-1* (*SLC7A1*), *CAT2-B* (*SLC7A2*)), neutral amino acid transporters (*ASCT1(SLC1A4)*) and transporters for both cationic and neutral amino acids (*b*^*0,+*^*AT (SLC7A9), ATB*^*0,+*^ (*SLC6A14*), *y*^+^*LAT2* (*SLC7A6*)) [79–81]. *ATB*^*0,+*^and *ASCT1* are significantly increased at peak lactation [80]. Our lab reported that either *EAAC1* or *EAAT3 (SLC1A1)* is the predominant anionic amino acid transporter and is significantly increased during lactation [82]. Cationic amino acid transporters *CAT-1*, *CAT2-B*, *y*^+^*LAT1* and y^+^LAT2 are widely expressed in the mammary gland and *y*^+^*LAT2* is significantly increased at peak lactation [82]. Neutral amino acid transporters *ASCT1, ASCT2, LAT2 (SLC7A8)* and *SNAT2 (SLC38A2)* are also expressed in the mammary gland [82]. Recently, a significant increase of *B*^*0,+*^*AT* and *CAT-4* were observed in swine mammary gland during the transition from colostrogenesis to lactation [83]. However, compared with mRNA expression of amino acid transporters between d 4 and 14 of lactation, different stage of lactation did not influence the abundance of these mRNA, except for *ATB*^*0,+*^*,* which was 28% lower on d 14 compared with d 4 of lactation. This indicates that the sow mammary gland significantly expresses amino acid transporters as early as 4 d [84]. Currently, there is little information regarding the location (apical surface, basolateral surface or cytoplasm) of amino acid transporter proteins within porcine and other species’ mammary glands.

**Table 2:** Predominant amino acids transporters identified in porcine mammary glands

Systems	Proteins	Genes	Substrates
Cationic amino acid transporters			
	CAT1	*SLC7A1*	Arg, Lys, His
	CAT2-B	*SLC7A2*	Arg, Lys, His
Neutral amino acid transporters			
	ASCT1	*SLC1A4*	Ala, Ser, Cys
	ASCT2	*SLC1A5*	Ala, Ser, Cys, Thr, Gln
	LAT2	*SLC7A8*	Leu, Ile, Val, Trp
	SNAT2	*SLC38A2*	Gly, Pro, Ala, Ser, Cys, Gln, Asn, Ser, Met
Cationic and neutral amino acid transporters			
	y^+^LAT1	*SLC7A7*	Lys, Arg, Gln, His, Met, Leu
	y^+^LAT2	*SLC7A6*	Lys, Arg, Gln, His, Met, Leu, Ala, Cys
	b^0,+^AT	*SLC7A9*	Arg, Lys, Cys
	ATB^0,+^	*SLC6A14*	All neutral and cationic amino acids
Anionic amino acid transporters			
	EAAT3	*SLC1A1*	Glu, Asp

Note: References reporting amino acid transporters in porcine mammary gland: [79–81]

Mammary gland epithelial cells transport free amino acids from blood through the basolateral surface and synthesize them into milk protein in the mammary gland. It has been reported that free amino acid concentrations in porcine milk are very low [85]. Therefore, we hypothesize the functional amino acid transporters should exist on the basolateral side. Consistent with this hypothesis, some amino acid transporters such as: y^+^LAT1/4F2hc and y^+^LAT2/4F2hc are expressed in the basolateral cell membrane in other organs [86, 87]. However, most of the amino acid transporters expressed in the mammary gland (ATB^0,+^, b^0,+^AT, CAT-1 and etc.) are localized only at the apical pole of cells in other organs (e.g., intestinal and kidney cells). This might due to diversity of the location of these amino acid transporters among different cell types. For example, EAAT3 was predominantly located in the basolateral cell membrane in pigmented epithelial cells, while it was mainly identified on the apical surface in Madin-Darby canine kidney cells [88]. Therefore, future research should use confocal immunofluorescence to localize these amino acid transporters. Furthermore, within 24 h after parturition, large amounts of immunoglobins are directly transferred from blood to milk instead of their components amino acids [89] . This process might rely on the neonatal Fc receptor (FcRn) expressed in the porcine mammary gland [90].

**Fig. 3:**
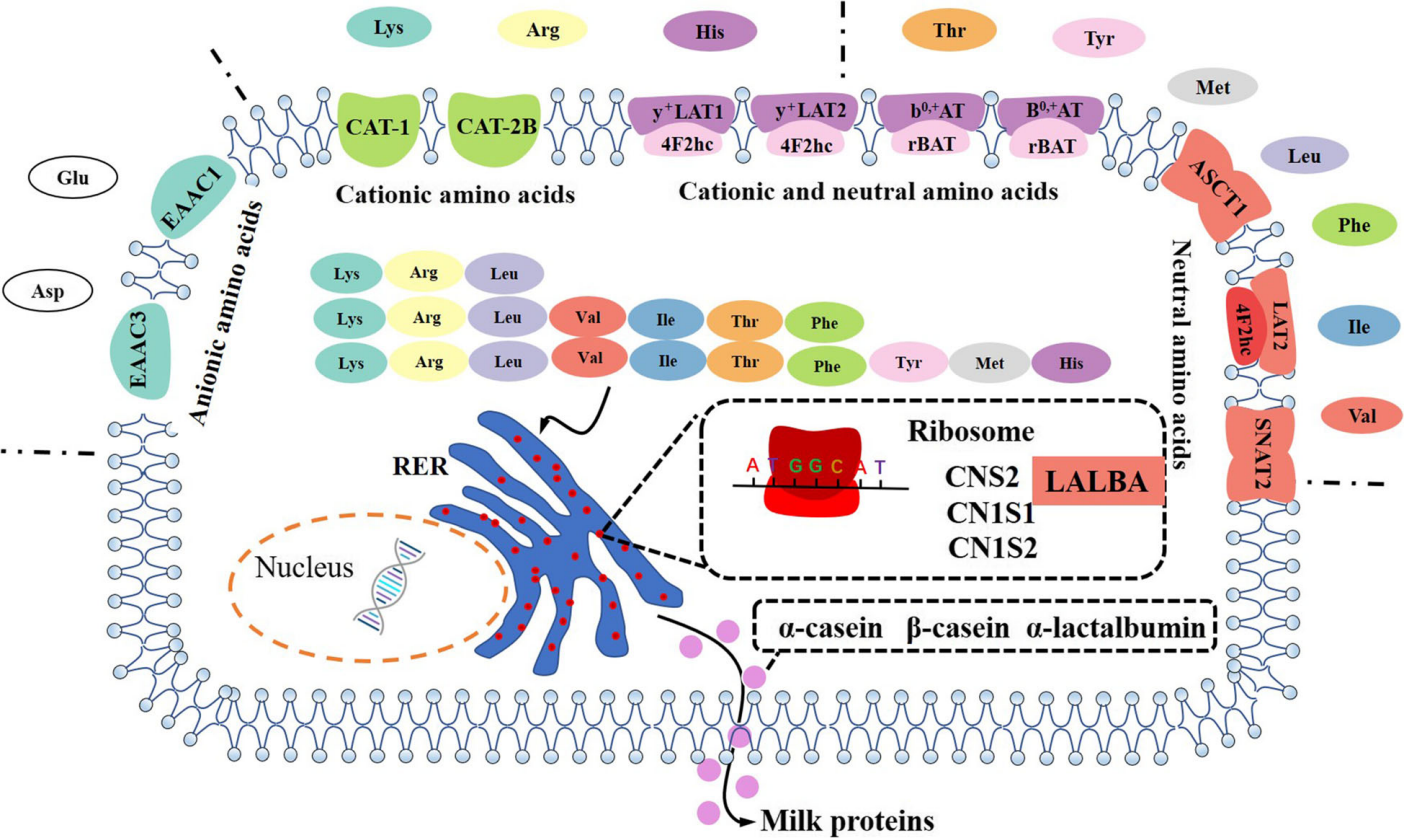
Milk protein synthesis pathway in porcine mammary gland. Note: Amino acids from blood are taken up by the porcine mammary gland through cationic amino acid transporters (CAT-1 and CAT-2B), neutral amino acid transporters (ASCT1, LAT2 and SNAT2), cationic and neutral amino acid transporters (b^0,+^AT, ATB^0,+^,y^+^LAT1, y^+^LAT2), and anionic amino acid transporters (EAAC1 and EAAC3). Amino acid uptake by the porcine mammary gland can be divided into three levels: high level (leucine, arginine and lysine), middle level (valine, isoleucine, threonine, phenylalanine), and low level (tyrosine, methionine and histidine). Subsequently, amino acids are converted into caseins and whey proteins, such as, α_S1_-casein (CSN1S1), α_S2_-casein (CSN1S2), β-casein (CSN2), κ-casein (CSN3), α-lactalbumin (LALBA), whey acidic protein (WAP), LTF (lactotransferrin), ALB (albumin). Abbreviations: CAT1, cationic amino acid transporter1; CAT-2B, cationic amino acid transporter 2B; ASCT1, system ASC neutral amino acid transporter 1; LAT2, L-type amino acid transporter 2; SNAT2, sodium-coupled neutral amino acid transporter 2; b^0,+^AT, b^0,+^ amino acid transporter; ATB^0,+^, B^0,+^amino acid transporter; y^+^LAT1, y^+^-type amino acid transporter 1; y^+^LAT2, y^+^-type amino acid transporter 2; EAAC1, excitatory amino-acid carrier 1; EAAC3, excitatory amino-acid carrier 3

**Fig. 4:**
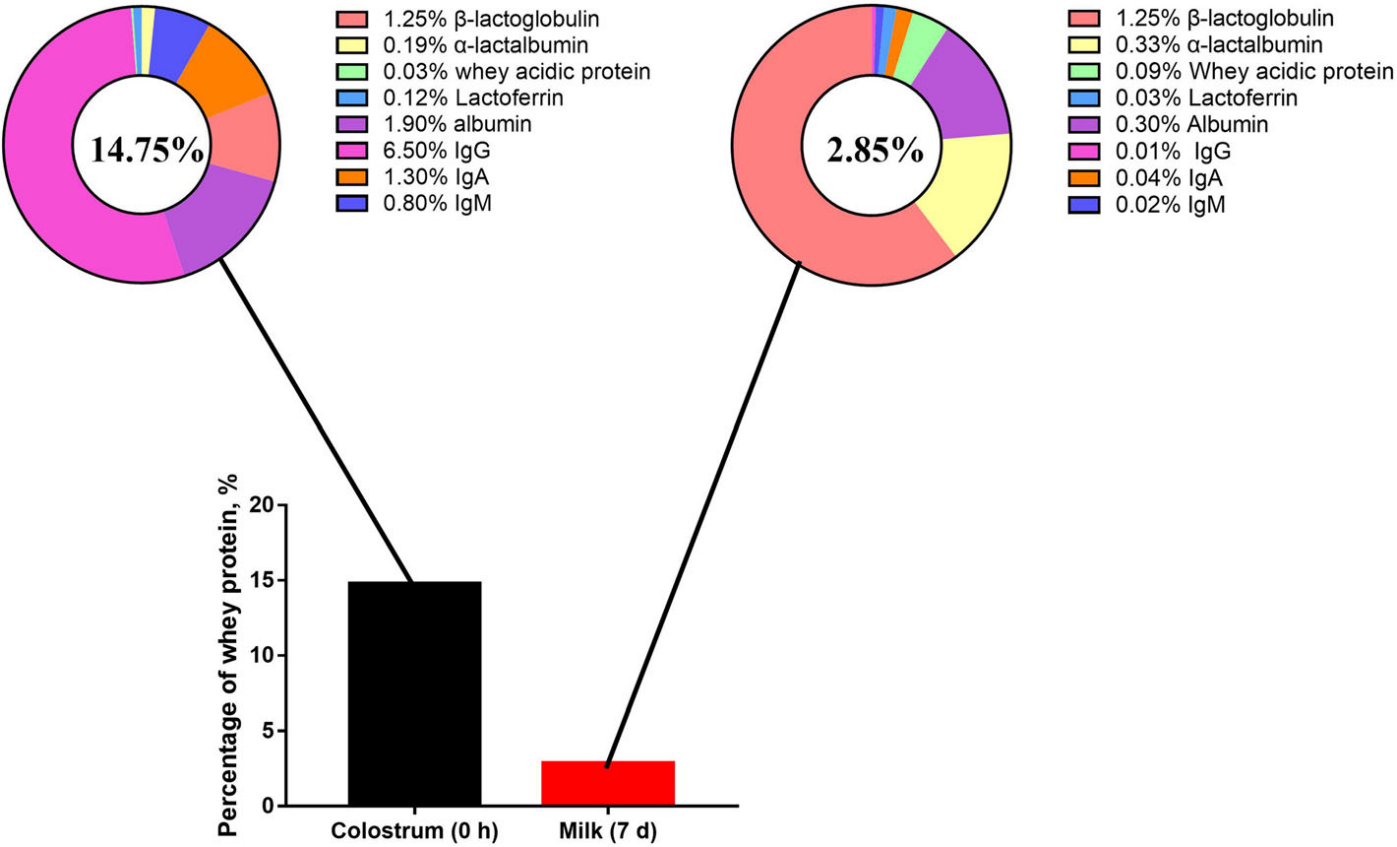
Components of whey protein in colostrum and milk. Note: Data are summarized from the information provided by Hurley [93]

After uptake of free amino acids from blood into the porcine mammary gland, they are synthesized into milk protein, including caseins and whey proteins. The caseins in porcine milk contain α_S1_-casein (CSN1S1), α_S2_-casein (CSN1S2), β-casein (CSN2), and κ-casein (CSN3) [91]. It has been demonstrated that *CSN2*, *CSN1S1* and *CSN1S2* are the most abundant three genes related to protein synthesis in the porcine mammary gland [92]. Except for caseins, the remaining proteins are whey proteins, which are mainly composed of β-lactoglobulin (BLG), α-lactalbumin (LALBA) and whey acidic protein (WAP) [91]. The possible mechanism of the amino acid transporter and protein synthesis system is summarized in Fig. 3. Recently, Hurley [93] comprehensively summarized reports of the concentrations of all the components of whey protein, which are listed in Fig. 4. Furthermore, maternal immunoglobulins also belong to milk whey proteins, and play a vital role in maternal immunity for neonates. The shift of immunoglobulin levels in the porcine mammary gland during the entirety of lactation period is well documented in previous research [94]. In colostrum, all IgG and nearly 80% of IgM are derived from the serum of the sow. Immune cells that migrate to the mammary gland contribute around 90% of IgA and IgM, and 70% IgG in milk [89].

### Fatty acid transportation

The mechanisms involved in fatty acid uptake by porcine mammary epithelial cells are still largely unknown. It was reported that cellular fatty acid uptake might be modulated by intracellular fatty acid-binding proteins (FABPs). Currently, 9 isoforms of fatty acid binding proteins have been identified [95]. FABP3 is predominantly expressed in bovine and dairy goat mammary glands during lactation compared with other FABP isoforms [96, 97]. However, FABP4 was reported to be significantly associated with fatty acid uptake in the bovine mammary gland [98]. Inhibition of FABP3 has been reported to reduce the synthesis of medium-chain fatty acids in the goat mammary gland [99]. Recently, FABP3 was detected in the porcine mammary gland and is primarily expressed during lactation [100]. However, Lv et al. [100] did not attempt to identify other FABP isoforms in the porcine mammary gland. We could not find any information that indicated that FABP3 is the only FABP isoform that exists in the porcine mammary gland. Therefore, future studies are needed to clarify other potential FABP isoforms in the porcine mammary gland. Milk from cattle contains short, medium and long chain fatty acids [101, 102] while fatty acid profiles in sow’s milk are mainly composed of long-chain fatty acids. The profiles of major fatty acids in sow colostrum and milk are listed in Table 3. The major fatty acids in sow colostrum and milk are C14:0, C16:0, C16:1 (*n*-7), C18:0, C18:1(*n*-9) and C18:2 (*n*-6). The literature reporting sow fatty acid concentrations is limited, but it is reasonable to conclude that sow milk contains relatively high levels of C14:0, C16:0 and C16:1 (*n*-7) and low levels of C18: 2(*n*-6) compared to colostrum.

**Table 3:** Major fatty acid profiles in colostrum and milk (mg/g)

Fatty acids	Colostrum		Milk	
	0 d	10 d	17 d	22 d
C14: 0	1.41	3.08	3.34	3.93
C16: 0	19.73	28.82	27.96	33.46
C16: 1	2.77	6.87	8.02	10.05
C18: 0	4.66	4.95	4.13	4.14
C18: 1	29.11	32.53	27.21	29.48
C18: 2(*n*-6)	20.91	22.01	13.20	17.52

Note: References used to calculate percentage of fatty acids: Bai et al. [106]; Amusquivar et al. [122]; Bee [123]

**Fig. 5:**
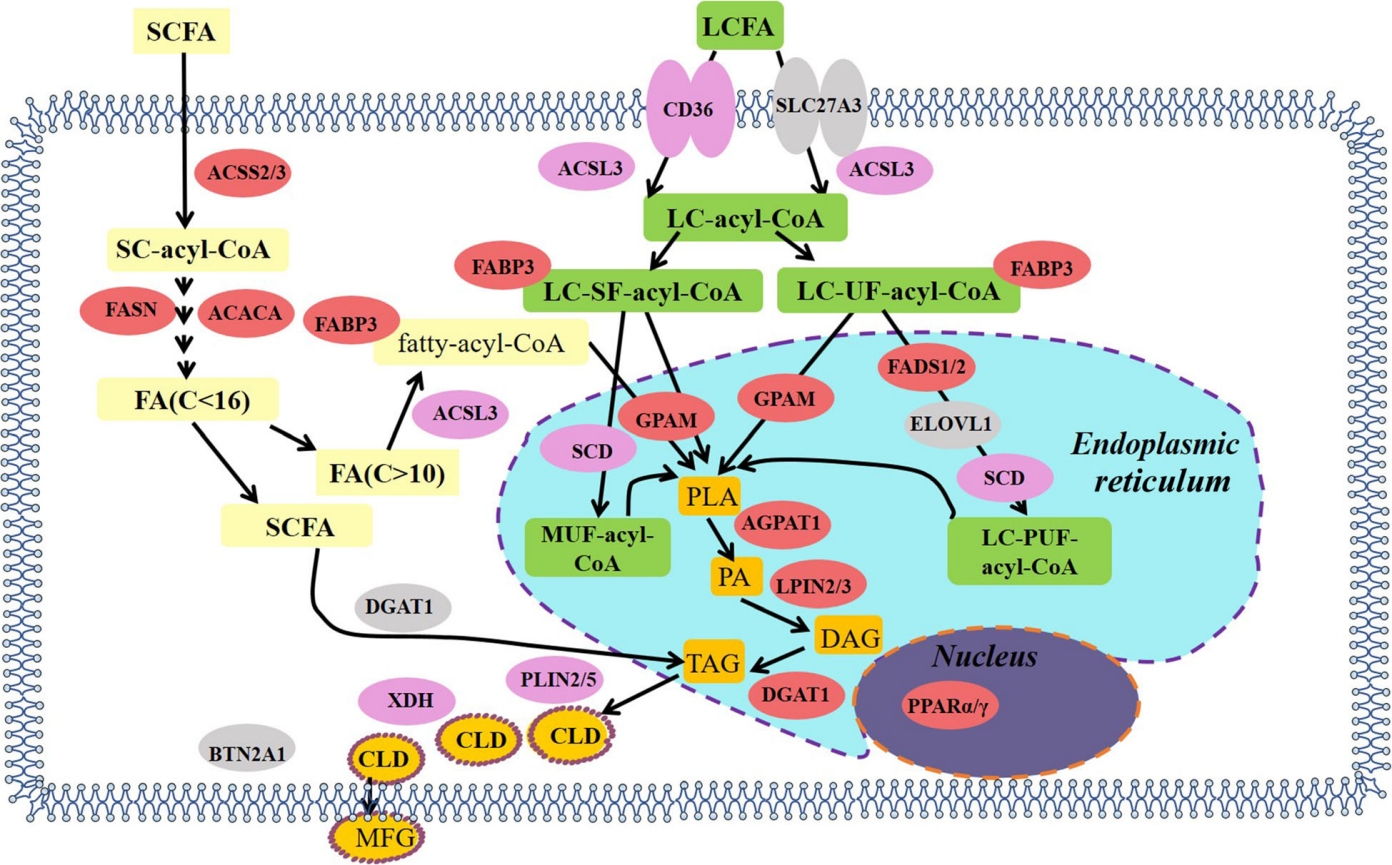
Lipid synthesis pathway in porcine mammary gland. Note: LCFA the enter porcine mammary gland, facilitated by transport proteins (mainly CD36), and are converted into their activated form LC-acyl-CoA, with the help of ACSL. Cytosolic LC-acyl-CoA is transported to the endoplasmic reticulum membrane by FABP3 and esterified there to glycerol-3-phosphate to produce LPA by GPAM. In the endoplasmic reticulum, PA can be hydrolyzed with LPIN to form DAG, which then is acylated to form TAG by DGAT. Newly-formed TAG forms cytoplasmic lipid droplets in the ER membrane via incorporation. The cytoplasmic lipid droplets are then transported to the apical membrane, and eventually released into porcine milk. A series of enzymes are required to facilitate this process, of which FASN and ACACA are considered the crucial enzymes of cellular fatty acid de novo synthesis. ACACA carboxylates acetyl-CoA to form malonyl-CoA, which is further converted by FASN to fatty acids (C ≤ 16). The synthesized fatty acids then participate in TAG formation. After the initation of lactation, mRNA of enzymes is increased 5-fold or more are shown in light purple; those increasing 2-fold or more are colored light red, and those expression increase or decrease less than 2-fold are shown in light gray. Abbreviations: ACACA, acetyl-CoA carboxylase alpha; ACSL3, acyl-CoA synthetase long-chain family member 3; ACSS2, acyl-CoA synthetase short-chain family member 2; AGPAT6, 1-acyl-sn-glycerol-3-phosphate acyltransferase 6; CD36, fatty acid translocase/CD36; CLD, cytoplasmic lipid droplet; DAG, diacylglycerol; DGAT1, diacylglycerol acyltransferase 1; FABP3, fatty acid binding protein 3; FASN, fatty acid synthase; GPAM, glycerol-3-phosphate acyltransferase, mitochondrial; LCFA, long chain fatty acid; LPA, lysophosphatidic acid; LPIN2, lipin 2; MFG, milk fat globule; PA, phosphatidic acid; PLIN2, perilipin 2; PPARγ, peroxisome proliferator-activated receptor γ; SCFA, short chain fatty acid; SCD, stearoyl-CoA desaturase; TAG, triacylglycerol

After uptake into the mammary gland, free fatty acids will be converted into triacylglycerol (TAG). The process of TAG synthesis in the porcine mammary gland was reported by Lv et al. [100]. They found some of the critical genes involved in milk TAG synthesis and secretion, including those related to FA uptake, FA activation, intracellular transport, de novo FA synthesis, FA elongation, FA desaturation, TAG synthesis, lipid droplet formation, transcription factors and nuclear receptors (Table 4). A proposed mechanism of milk fat synthesis is summarized in Fig. 5.

**Table 4:** Critical genes involved in fatty acid synthesis in porcine mammary gland

Functions	Genes
FA uptake	Very low density lipoprotein receptor, lipoprotein Lipase, fatty acid translocase/CD36
FA activation	Acyl-CoA synthetase short-chain family member 2, Acyl-CoA synthetase long-chain family member 3
Intracellular transport	Fatty acid-binding protein 3
De novo FA synthesis	Acetyl-CoA carboxylase alpha, fatty acid synthase
FA elongation	Elongation of very-long-chain fatty acids 1
FA desaturation	Stearoyl-CoA desaturase, fatty acid desaturase 1
TAG synthesis	Glycerol-3-phosphate acyltransferase, 1-acyl-sn-Glycerol-3-phosphate acyltransferase 1, lipin 1, Diacylglycerol acyltransferase 1
Lipid droplet formation	Butyrophilin, dehydrogenase, adipophilin 2
Transcription factors and nuclear receptors	Sterol response element binding protein 1, Cleavage-activating protein, insulin induced gene 1 or 2

## Conclusion

Porcine milk is a highly complicated bio-fluid that nourishes neonates and protects them from pathogens and disease. In the past 30 years, the concentrations of major components (protein, fat and lactose) in colostrum seem quite stable and only minor changes were observed in fat and lactose concentrations in mature milk. Beside major nutrient components, recent research reported other vital bioactive components (oligosaccharides, exosomes, and bacteria) in porcine milk. These antimicrobial and immunomodulatory components of porcine milk are hypothesized to compensate for immature neonatal immune systems and mitigate environmental infectious pathogens. Understanding the nutritional and non-nutritional components in porcine milk is critical for efficient pig production. However, information and effects regarding these bioactive components in in vivo pig model is still limited. Some vital questions should be addressed in the future: 1) How do sow genotypes affect sow milk composition? 2) Do different oligosaccharides regulate the bacterial biome and immunity in the GI tract of piglets and protect sows’ mammary glands from bacterial infection? 3) What is the source of the bacteria in milk, gut migration or contamination from milking? 4) Do exosomes regulate piglets’ gut function?

Recent studies provide some preliminary data on the nutritional transporter and synthesis systems associated with the porcine mammary gland. Cited reports in this review provide some information on the expression of glucose, amino acid and fatty acid transporters and document potential milk lactose, protein and fat synthesis pathways in the mammary gland. In order to further study these nutritional transporters, these transporters need localized with immunofluorescence microscopy or confocal microscopy. Additionally, the biological effects of non-coding RNA (miRNA, circular RNA) on mammary gland transporter and synthesis systems are largely unknown. Understanding these nutrition transporter and synthesis systems might provide a rational approach to regulate milk composition in the future.

## Additional file

Additional file 1: **Table S1.** Milk composition of different species (%). **Table S2.** Averages and range of reported concentrations of protein, fat and lactose of sow colostrum in 2010s. **Table S3.** Averages and range of reported concentrations of protein, fat and lactose of sow milk in 2010s. (DOCX 31 kb)

## Abbreviations

ACACA: Acetyl-CoA carboxylase alpha
ACSL3: Acyl-CoA synthetase long-chain family member 3
ACSS2: Acyl-CoA synthetase short-chain family member 2;
AGPAT6: 1-acyl-sn-glycerol-3-phosphate acyltransferase 6
ASCT1: System ASC neutral amino acid transporter 1
ATB^0,+^: B^0,+^amino acid transporter;
b^0,+^AT: b^0,+^ amino acid transporter;
B4GALT: Beta-1, 4-galactosyl transferase
CAT1: Cationic amino acid transporter1
CAT-2B: Cationic amino acid transporter 2B;
CD36: Fatty acid translocase/CD36
CLD: Cytoplasmic lipid droplet
DAG: Diacylglycerol
DGAT1: Diacylglycerol acyltransferase 1
EAAC1: Excitatory amino-acid carrier 1
EAAC3: Excitatory amino-acid carrier 3
FABP3: Fatty acid binding protein 3
FASN: Fatty acid synthase
GALE: UDP-glucose 4-epimerase gene
GALT: Galactose-1-phosphate uridyltransferase
GLUT: Glucose transporter
GPAM: Glycerol-3-phosphate acyltransferase, mitochondrial
LALBA: Alpha-lactalbumin gene β1,4-galactosyltransferase.
LAT2: L-type amino acid transporter 2
LCFA: Long chain fatty acid
LPA: Lysophosphatidic acid
LPIN2: Lipin 2
MFG: Milk fat globule
PA: Phosphatidic acid
PGM: Phosphoglycerate mutase
PLIN2: Perilipin 2
PMOs: Porcine milk oligosaccharides
PPARγ: Peroxisome proliferator-activated receptor γ
SCD: Stearoyl-CoA desaturase
SCFA: Short chain fatty acid
SGLT: Sodium-dependent glucose cotransporter
SNAT2: Sodium-coupled neutral amino acid transporter 2
TAG: Triacylglycerol
UGP2: UDP-glucose pyrophosphorylase 2
y^+^LAT1: y^+^-type amino acid transporter 1
y^+^LAT2: y^+^-type amino acid transporter 2
