# Supplementary material for: Recent progress of porcine milk components and mammary gland function
Source: J Anim Sci Biotechnol. 2018 Oct 22;9:77. doi: 10.1186/s40104-018-0291-8 (PMC6196465; doi:10.1186/s40104-018-0291-8)
Supplement: Supplementary file 1 — Table S1. Milk composition of different species (%). Table S2. Averages and range of reported concentrations of protein, fat and lactose of sow colostrum in 2010s. Table S3. Averages and range of reported concentrations of protein, fat and lactose of sow milk in 2010s. (DOCX 31 kb) [file 40104_2018_291_MOESM1_ESM.docx]

**Table S1** Milk composition of different species (%)^1^

| Specious | Fat | Protein | Lactose | Ash | Total solids |
| --- | --- | --- | --- | --- | --- |
| Antelope | 1.3 | 6.9 | 4 | 1.3 | 25.2 |
| Ass (donkey) | 1.2 | 1.7 | 6.9 | 0.45 | 10.2 |
| Bear, polar | 31 | 10.2 | 0.5 | 1.2 | 42.9 |
| Bison | 1.7 | 4.8 | 5.7 | 0.96 | 13.2 |
| Buffalo, Philippine | 10.4 | 5.9 | 4.3 | 0.8 | 21.5 |
| Camel | 4.9 | 3.7 | 5.1 | 0.7 | 14.4 |
| Cat | 10.9 | 11.1 | 3.4 | --- | 25.4 |
| Cow: |  |  |  |  |  |
| Ayrshire | 4.1 | 3.6 | 4.7 | 0.7 | 13.1 |
| Brown Swiss | 4 | 3.6 | 5 | 0.7 | 13.3 |
| Guernsey | 5 | 3.8 | 4.9 | 0.7 | 14.4 |
| Holstein | 3.5 | 3.1 | 4.9 | 0.7 | 12.2 |
| Jersey | 5.5 | 3.9 | 4.9 | 0.7 | 15 |
| Zebu | 4.9 | 3.9 | 5.1 | 0.8 | 14.7 |
| Deer | 19.7 | 10.4 | 2.6 | 1.4 | 34.1 |
| Dog | 8.3 | 9.5 | 3.7 | 1.2 | 20.7 |
| Dolphin | 14.1 | 10.4 | 5.9 | --- | 30.4 |
| Elephant | 15.1 | 4.9 | 3.4 | 0.76 | 26.9 |
| Goat | 3.5 | 3.1 | 4.6 | 0.79 | 12 |
| Guinea Pig | 3.9 | 8.1 | 3 | 0.82 | 15.8 |
| Horse | 1.6 | 2.7 | 6.1 | 0.51 | 11 |
| Human | 4.5 | 1.1 | 6.8 | 0.2 | 12.6 |
| Kangaroo | 2.1 | 6.2 | Trace | 1.2 | 9.5 |
| Mink | 8 | 7 | 6.9 | 0.7 | 22.6 |
| Monkey | 3.9 | 2.1 | 5.9 | 2.6 | 14.5 |
| Opossum | 6.1 | 9.2 | 3.2 | 1.6 | 24.5 |
| Pig | 8.2 | 5.8 | 4.8 | 0.63 | 19.9 |
| Rabbit | 12.2 | 10.4 | 1.8 | 2 | 26.4 |
| Rat | 14.8 | 11.3 | 2.9 | 1.5 | 31.7 |
| Reindeer | 22.5 | 10.3 | 2.5 | 1.4 | 36.7 |
| Seal, gray | 53.2 | 11.2 | 2.6 | 0.7 | 67.7 |
| Sheep | 5.3 | 5.5 | 4.6 | 0.9 | 16.3 |
| Whale | 34.8 | 13.6 | 1.8 | 1.6 | 51.2 |

^1^These data were summarized by [Jensen [9]](#_ENREF_9).

**Table S2** Averages and range of reported concentrations of protein, fat and lactose of sow colostrum in 2010s

| Colostrum | Time from parturition (h) | | | | |
| --- | --- | --- | --- | --- | --- |
|  | 0 | 1 | 3 | 6 | 24 |
| Protein, % |  |  |  |  |  |
| Averages | 16.6 | 16.1 | 14.3 | 15.3 | 8.5 |
| Range | 16.4-16.8 | 7.7-20.2 | - | - | 7.14-10.16 |
| Studies | 2 | 3 | 1 | 1 | 3 |
| Fat, % |  |  |  |  |  |
| Averages | 5.3 | 5.7 | 5.75 | 5.2 | 8.07 |
| Range | 5.2-5.4 | 4.3-7.2 | - | - | 7.66-8.3 |
| Studies | 2 | 3 | 1 | 1 | 3 |
| Lactose, % |  |  |  |  |  |
| Averages | 2.9 | 3.1 | 5.15 | 2.5 | 3.25 |
| Range | 2.5-3.3 | 1.6-4.2 | - | - | 2.25-4.1 |
| Studies | 2 | 3 | 1 | 1 | 3 |

**Table S3** Averages and range of reported concentrations of protein, fat and lactose of sow milk in 2010s

| Milk | Day of lactation (d) | | | | | | |
| --- | --- | --- | --- | --- | --- | --- | --- |
|  | 2 | 3 | 7 | 10 | 14 | 17 | 21 |
| Protein, % |  |  |  |  |  |  |  |
| Averages | 8.56 | 5.83 | 5.04 | 4.99 | 4.83 | 4.6 | 4.81 |
| Range | 8.41-8.71 | 5.2-6.9 | 3.68-6.09 | 3.66-6.12 | 4.1-5.23 | 4.5-4.7 | 3.5-6.1 |
| Studies | 2 | 3 | 4 | 7 | 4 | 2 | 9 |
| Fat, % |  |  |  |  |  |  |  |
| Averages | 7.48 | 8.58 | 7.84 | 7.75 | 7.33 | 6.45 | 7.04 |
| Range | 5.48-9.47 | 6.7-10.24 | 6.69-8.7 | 6.41-10.1 | 6.65-8.4 | 6.1-6.8 | 5.45-10.5 |
| Studies | 2 | 3 | 4 | 7 | 4 | 2 | 9 |
| Lactose, % |  |  |  |  |  |  |  |
| Averages | 3.41 | 4.68 | 4.11 | 5.11 | 5.53 | 5.45 | 5.29 |
| Range | 3.33-3.48 | 4.03-5.2 | 2.32-6.4 | 3.5-6.3 | 5.18-5.7 | 5.2-5.7 | 3.8-6.4 |
| Studies | 2 | 3 | 4 | 7 | 4 | 2 | 9 |
